# Keeping 3D electron crystallography relevant: Leveraging new technologies for 3D ED/MicroED at a regional cryoEM center

**DOI:** 10.1063/4.0000955

**Published:** 2025-10-27

**Authors:** M. Jason de la Cruz

**Affiliations:** Structural Biology Program, Sloan Kettering Institute, Memorial Sloan Kettering Cancer Center, New York, NY, USA; Cryo-Electron Microscopy Innovation Laboratory, Memorial Sloan Kettering Cancer Center, New York, NY, USA

## Abstract

The crystallographic method MicroED (3D ED/MicroED) has seen a resurgence for protein structure determination since 2013, and for solving small-molecule structures since 2018. However, since the cryoEM “resolution revolution” in 2014, there has not been widespread adoption of the technique at cryoEM facilities as compared with single particle analysis. I discuss my experiences concerning the setup and use of MicroED at the New York Structural Biology Center, from 2018 to present, as well as technological advances in the cryoEM technique since then. In particular, I highlight how these improvements have guided current development and use of MicroED in multiuser facilities.

